# Antivirals Targeting the Surface Glycoproteins of Influenza Virus: Mechanisms of Action and Resistance

**DOI:** 10.3390/v13040624

**Published:** 2021-04-06

**Authors:** Yaqin Bai, Jeremy C. Jones, Sook-San Wong, Mark Zanin

**Affiliations:** 1 State Key Laboratory of Respiratory Diseases, Guangzhou Medical University, 195 Dongfengxi Rd, Guangzhou 510182, China; Sarah_By@163.com (Y.B.); sook-san.wong@gird.cn (S.-S.W.); 2 Department of Infectious Diseases, St. Jude Children’s Research Hospital, 262 Danny Thomas Place, Memphis, TN 38105, USA; Jeremy.Jones@STJUDE.ORG; 3 School of Public Health, The University of Hong Kong, Hong Kong, China

**Keywords:** Influenza virus, hemagglutinin, neuraminidase, antiviral resistance, antiviral

## Abstract

Hemagglutinin and neuraminidase, which constitute the glycoprotein spikes expressed on the surface of influenza A and B viruses, are the most exposed parts of the virus and play critical roles in the viral lifecycle. As such, they make prominent targets for the immune response and antiviral drugs. Neuraminidase inhibitors, particularly oseltamivir, constitute the most commonly used antivirals against influenza viruses, and they have proved their clinical utility against seasonal and emerging influenza viruses. However, the emergence of resistant strains remains a constant threat and consideration. Antivirals targeting the hemagglutinin protein are relatively new and have yet to gain global use but are proving to be effective additions to the antiviral repertoire, with a relatively high threshold for the emergence of resistance. Here we review antiviral drugs, both approved for clinical use and under investigation, that target the influenza virus hemagglutinin and neuraminidase proteins, focusing on their mechanisms of action and the emergence of resistance to them.

## 1. Introduction

Vaccination remains one of the central public health interventions to combat seasonal influenza. However, vaccine development lead times of at least six months limit their applicability during outbreaks of novel influenza viruses. Antiviral drugs are useful interventions against novel influenza viruses, and they can reduce disease burden caused by seasonal strains. The influenza virus presents several targets for effective antivirals. The first class of anti-influenza drug to be approved for use were the adamantanes, which block the M2 ion channel on the surface of the virion [1]. This activity decreases ion flow and endosome acidification, inhibiting the low pH-induced fusion of the viral membrane with the endosome. Whilst these drugs were effective, the relatively rapid emergence of resistant strains ultimately abrogated their efficacy. Whilst M2 inhibitor stockpiles exist to treat sensitive strains, the use of these drugs was advised against by the United States Centers for Disease Control and Prevention (CDC) in 2006 [2]. Polymerase and nucleoprotein inhibitors that target the replication machinery of influenza viruses have also been developed. Two of these drugs, baloxavir marboxil and favipiravir, have reached the market but are not commonly used at present. Baloxavir marboxil is under license in Japan, the United States of America (USA), Hong Kong, Australia and Europe, whilst favipiravir is licensed for limited use in Japan and under Phase III clinical trial in the USA and Europe [3].

The surface glycoproteins of influenza A and B viruses, consisting of the hemagglutinin (HA) and neuraminidase (NA) proteins, are the most exposed proteins of influenza virus. This makes them prominent targets for interventions such as vaccines, antibodies and antivirals. They can be thought of as having opposing roles, in that HA mediates cell attachment and infection and NA mediates the release of progeny virions from cells by enzymatically releasing viruses from the surface of progeny cells. HA inhibitors interfere with the attachment of HA to sialic acids on the surface of the cells or with the conformational changes that occur in HA that mediate viral fusion and release of the viral genome into the cell. NA inhibitors (NAIs) block the sialidase activity of NA, preventing the release of progeny virions. The most commonly used class of antiviral against influenza viruses are the NAIs. Inhibitors targeting HA are relatively new and have not been applied clinically to the same extent as NAIs. Whilst NAIs remain an effective intervention against influenza viruses, the development of resistance remains a concern, as it does for HA inhibitors. The focus of this review is antiviral resistance to HA and NA inhibitors from the perspectives of epidemiology, molecular virology and pharmacology with the goal of providing a review of the impact, developments and challenges associated with antiviral drug resistance in influenza viruses.

## 2. Neuraminidase Inhibitors

**Figure 1:**
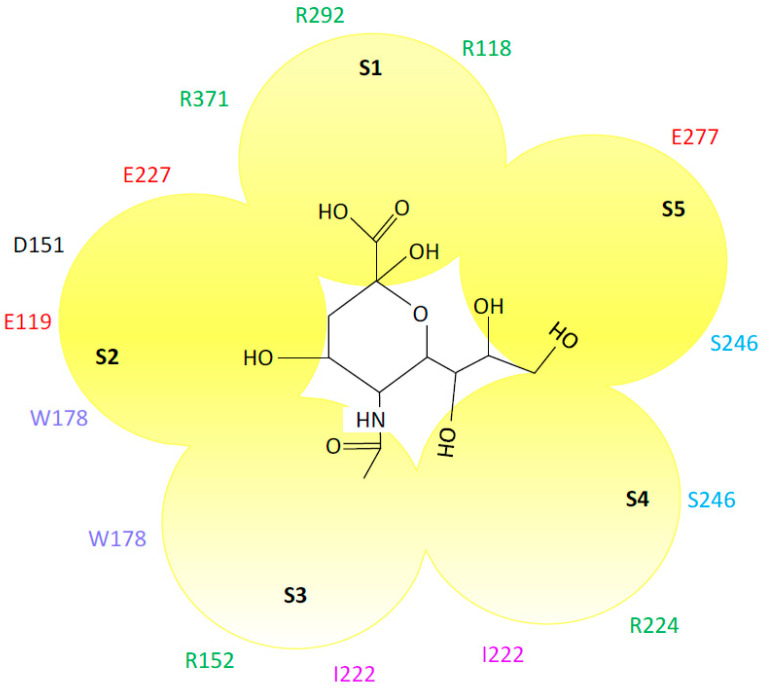
Interaction of the neuraminidase active site with sialic acid. The interacting amino acids and the active site subsites (S1–S5) are shown (N2 numbering). Glutamic acid (red), serine (blue), arginine (green), tryptophan (violet) and aspartic acid (black). Sialic acid is shown in the center of the figure. Adapted from References [8,9]. Sialic acid structure was adapted from PubChem.

NA is a sialidase that binds and cleaves N-acetyl-neuraminic acid, releasing progeny virions from the cell surface. NA is expressed on the surface of the influenza A and B viruses as a homotetrameric glycoprotein. Each monomer consists of four distinct structural domains; the catalytic head containing the active site, the stalk, the transmembrane region and the cytoplasmic tail [4,5]. The active sites are composed of 19 highly conserved residues, eight catalytic residues that directly interact with sialic acids (R118, D151, R152, R224, E276, R292, R371, and Y406) and 11 framework residues that maintain the structure of the active site (E119, R156, W178, S179, D198, I222, E227, H274, E277, N294, and E425) (N2 numbering, used throughout) [5]. The active site can be divided into five subsites: S1 to S5 [6,7,8]. R118, R292, and R371 comprise S1; W178, E119, D151, and E227 comprise S2; R152, W178, and I222 comprise S3; I222, R224, and S246 comprise S4; and S246 and E277 comprise S5 (Figure 1) [9]. S1 interacts with the carboxylate side chain of sialic acids, R152 binds to the acetamido group on the sugar ring, and E276 interacts with the 8- and 9-hydroxyl groups on the glycerol side chain [5]. As the active site is highly conserved in both amino acid sequence and spatial orientation among influenza A and B viruses and is prominently located on the surface of the virion, it is a useful target for antiviral therapy.

### 2.1. Neuraminidase Inhibitors—Mechanisms of Action

**Figure 2:**
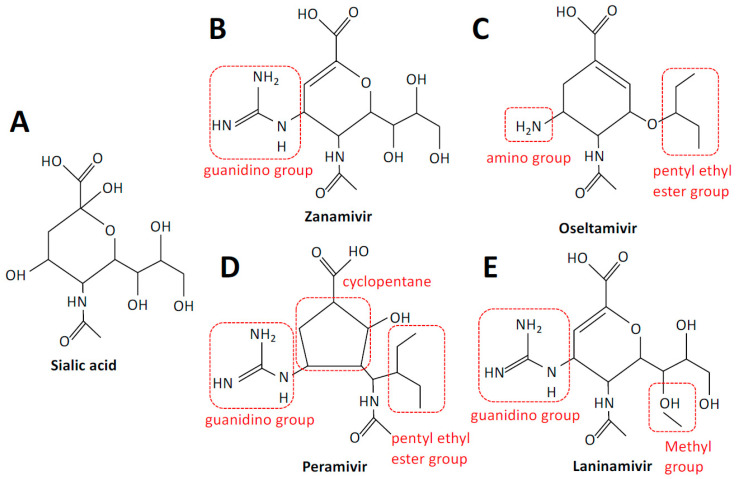
Structures of sialic acid and neuraminidase inhibitors. Sialic acid (**A**), zanamivir (**B**), oseltamivir (**C**), peramivir (**D**), and laninamivir (**E**). Structural elements are highlighted in red. Structures were adapted from PubChem.

NAIs are designed to mimic the substrate of NA yet bind with higher affinity. There are four NAIs in use: zanamivir, oseltamivir, peramivir, and laninamivir. Zanamivir (4-guanidino-Neu5Ac2en), oseltamivir, and peramivir (DB06614) are derivatives of the earliest transition state analog of the NA substrate N-acetylneuraminic acid (2,3-dehydro-2-deoxy-N-acetylneuraminic acid, DANA), whilst laninamivir (R125489) was produced via a modification of zanamivir. Two NAIs are licensed worldwide: zanamivir and oseltamivir phosphate (GS-4104). Peramivir is licensed in Japan, South Korea, China, and the USA [10,11], while laninamivir is only licensed in Japan but is currently in Phase III clinical trials in the USA (Table 1 and Figure 2). In the USA, zanamivir and oseltamivir were approved for use in 1999, and peramivir was licensed in 2014. Laninamivir was licensed for use in Japan in 2010.

Zanamivir is an analog of DANA, with a positively charged guanidino group replacing a hydroxyl group linked to C-4, which increases binding approximately 10,000-fold [12]. Zanamivir has a low bioavailability of 2%, necessitating delivery via inhalation. Inhalation of powder leads to approximately 7 to 21% of a drug to be deposited in the lower respiratory tract and the remainder in the oropharynx [13]. An intravenous formulation has also been developed for compassionate use only [14]. Zanamivir is well tolerated and approved for the prevention and treatment of acute, uncomplicated influenza in ambulatory adults and children. The dosing regimen for adults is typically two inhalations at 10 mg twice a day for five days, for therapy, and once a day for ten days, for prophylaxis.

Oseltamivir carboxylate (GS-4071) differs from DANA in that the C4-OH and glycerol side chains of DANA are replaced by an amine group and a pentyl ether side chain, respectively. Oseltamivir phosphate (GS-4014) was developed as a prodrug of oseltamivir carboxylate, to improve bioavailability, which is greater than 80% when administered orally [15]. It is indicated for the prevention of influenza A and B virus infection in patients ≥1 year of age and administered once per day; however, oseltamivir has been shown to be safe in children as young as two weeks of age. In adults, the recommended dosing regimen for therapy is 75 mg twice per day or 75 mg once per day for prophylaxis. Oral oseltamivir is generally well tolerated.

Peramivir is more structurally distinct, as it is a cyclopentane derivative; however, structural components are shared with other NAIs, namely a cyclopentane ring with a guanidino group and a pentyl ether side chain as per oseltamivir and zanamivir, respectively. Poor performance following oral delivery necessitated an intravenous formulation of peramivir, making it the only approved intravenous therapy for influenza [16]. Peramivir is available as 150 and 300 mg solutions outside the USA and 200 mg solutions in the USA. It is approved as a single-dose infusion for the treatment of uncomplicated influenza in otherwise healthy adults, as a single dose was found to be insufficient in severely ill patients.

Laninamivir was produced via methylation of the C-7 hydroxyl group of zanamivir. It is administered via intranasal inhalation as the prodrug laninamivir octanoate (CS-8958), which is hydrolyzed to the active form after adsorption in the respiratory epithelium [17]. A single 20 mg dose administered daily for two days is recommended for prophylaxis, whilst a single 40 or 20 mg dose is recommended for treatment in patients ≥10 or <10 years of age, respectively.

Whilst NAI structures are based on DANA or designed to mimic its binding to NA, their differing structures result in interactions with different amino acids, and, consequently, resistance to one NAI does not necessarily confer resistance to others, although some mutations confer resistance to multiple NAIs. Oseltamivir binds to E119, D151, R152, R292, R371, and Y406 [18], whilst zanamivir interacts with R118, R292, R371, D151, R152, and E276, catalytic residues in the active site [19,20]. The negatively charged carboxylate group forms strong hydrogen bonds with R118, R292, and R371, while the methyl group of the acetamido binds the hydrophobic pocket formed by W178 and I222 [10]. The guanidino group forms stable hydrogen bonds and electrostatic interactions with the acidic groups of E119, D151, and E227 [21].

### 2.2. Epidemiology of NAI Resistance

Global surveillance of NAI susceptibility of influenza A and B viruses has been conducted by the World Health Organization (WHO) Global Influenza Surveillance and Response System (GISRS) Expert Working Group for Surveillance of Antiviral Susceptibility since the 2012–2013 season. In this season the frequency of NAI resistance in A(H1N1)pdm09 and A(H3N2) viruses was 0.9% and 0.4%, respectively, and 0.3% and 1.0% in B/Yamagata and B/Victoria viruses, respectively [22]. However, in the 2013–2014 season, 3.4% of A(H1N1)pdm09 and 0.3% of A(H3N2)viruses showed NAI resistance, with NAI resistance detected in 0.3% and 2.0% of Yamagata- and Victoria-lineage influenza B viruses, respectively [23]. The proportion of NAI-resistant viruses subsequently decreased and has remained relatively low since 2014 (in 2014–2015, A(H1N1)pdm09 (0.5%), A(H3N2) (0.2%), B/Yamagata-lineage (1.0%), and B/Victoria-lineage (0.7%); in 2015–2016, A(H1N1)pdm09 (1.8%), A(H3N2) (0.2%), B/Yamagata-lineage (0.4%), and B/Victoria-lineage (0.5%); in 2016–2017, A(H1N1)pdm09 (0.5%), A(H3N2) (0.1%), B/Yamagata-lineage (0.2%), and B/Victoria-lineage (0.4%); in 2017-2018, A(H1N1)pdm09 (1.5%), A(H3N2) (0.4%), B/Yamagata-lineage (0.6%), and B/Victoria-lineage (1.1%)) [24,25,26,27]. NAI resistance is more common among type-A viruses, compared to type-B viruses (Table 2) [28].

#### 2.2.1. Zanamivir

Resistance to zanamivir has been rare, likely due to the conformational similarity of zanamivir to DANA and its infrequent use [53,54,55]. Further, H274Y does not affect zanamivir binding to NA. Resistance to zanamivir, mediated by Q136K, has been detected in <2.3% of seasonal A(H1N1) and A(H3N2) viruses and sporadically in A(H1N1)pdm09 viruses [56,57,58]. Overall, seasonal strains remain sensitive to zanamivir [59].

#### 2.2.2. Oseltamivir

The first reports of influenza viruses with reduced sensitivity to oseltamivir mediated by H274Y were made in 1999 to 2002 [60]. However, these isolates were rare [60,61]. Resistant isolates were not detected during the 2004–2005 influenza season, and in the 2005–2006 and 2006–2007 seasons, isolation rates were very low, at 0.4% and 0.6%, respectively [62]. Findings in animal models demonstrating that oseltamivir-resistance was associated with reduced viral fitness indicated that these viruses would not be an important clinical issue [63]. However, oseltamivir resistance mediated by H274Y began emerging in Europe during the 2007–2008 influenza season in A/Brisbane/59/2007 (H1N1)-like viruses. These viruses quickly spread, becoming the predominant circulating strain globally within months [28,53]. In contrast to earlier strains in which H274Y negatively impacted viral fitness, the A/Brisbane/59/2007 (H1N1)-like viruses retained their overall fitness, largely due to permissive mutations in NA such as R194G, R222Q, V234M, and D344N [64,65]. During the 2008–2009 influenza season, a very high proportion of seasonal A(H1N1) influenza viruses resistant to oseltamivir were detected, in some cases greater than 90% [66,67]. Globally, approximately 15% of A(H1N1) influenza virus isolates showed resistance to oseltamivir; however, resistance was not detected in A(H3N2) influenza viruses. These isolates were primarily found in children and the immunocompromised who were receiving oseltamivir. This is likely due to prolonged periods of viral replication, higher viral loads and potentially sub-optimal doses of oseltamivir [45,64,68]. However, resistant isolates were also found in the absence of oseltamivir treatment, which is indicative of transmission of resistant viruses [42].

The emergence of the A(H1N1)pdm09 influenza viruses changed the landscape of oseltamivir resistance. Unlike the seasonal A(H1N1) strains that were circulating, these viruses were sensitive to oseltamivir, with resistance detected in <1.5% of isolates initially. Resistant strains were detected primarily in children of one to five years of age and the immunosuppressed and detected >10 days post-commencement of oseltamivir treatment [28,53]. In addition to H274Y, I223K, I223R, and/or G147R were also found in isolates from these patients [25,69,70]. By 2011, oseltamivir resistance in A(H1N1)pdm09 viruses was 1.6% globally, with increasing numbers of isolates obtained from patients with no oseltamivir treatment. In the 2014–2015 influenza season, resistance rates rose to 1.9% but then fell to 0.6% in the 2014–2015 influenza season and remained low, at 0.5–3.4% [25,71]. Following the 2009 pandemic, A/Brisbane/59/2007 (H1N1)-like viruses that showed such high rates of oseltamivir resistance were replaced as seasonal A(H1N1) viruses and have not reappeared. Unlike A(H1N1) subtypes, resistance in A(H3N2) subtype viruses remains relatively rare, at <0.5%, occurring mainly in young children and the immunocompromised [28,71].

#### 2.2.3. Peramivir and Laninamivir

Mutations that confer resistance to oseltamivir also confer resistance to peramivir, such as E119D/G, S246R, and H274Y in A(H1N1)pdm09 clinical isolates [36,53,72]. Further, 3.2% of A(H1N1)pdm09 isolates collected between 2009 and 2012 were resistant to peramivir, but not to laninamivir [36]. Laninamivir resistance has been observed in clinical isolates of A(H1N1)pdm09 and influenza B viruses, conferred by E119D/G, S246R, or R152K; however, resistance to laninamivir does not appear to be common or widespread [31,73].

### 2.3. Functional Neuraminidase Inhibitor Resistance Motifs

#### 2.3.1. Zanamivir

##### Resistance in Influenza A Viruses

Zanamivir contains a guanidino group that interacts with the conserved E119 residue in the active center pocket of NA [74]. As such, E119G is most commonly associated with zanamivir resistance, although E119A and E119D have also been reported to mediate resistance to other NAIs [30,33,74]. E119G confers resistance specific to zanamivir, whilst E119D affects binding of all NAIs. E119V, however, confers oseltamivir resistance without affecting the binding of zanamivir [32,75,76]. In an A(H1N1)pdm09 virus background, I222K/R also conferred reduced susceptibility to zanamivir [56,77,78]. Q136K/R, which confers resistance to zanamivir, peramivir, and laninamivir, has been detected in seasonal pre-pandemic A(H1N1), A(H1N1)pdm09 and seasonal A(H3N2) influenza viruses [79]. Most of these mutations are detected sporadically and are often only detected after laboratory passaging of the clinical isolate, indicating that viruses with this mutation were not the dominant population in the patient and arose, or were selected for, during virus culturing in vitro [79].

##### Resistance in Influenza B Viruses

Zanamivir resistance has been found in B/Victoria- and B/Yamagata-lineage viruses across influenza seasons at relatively low incidence. Resistance has been associated with several mutations in clinical specimens such as E105K/E, E117G, H134N, D197N/E, A200T, and I221T (B lineage specific numbering) [22,23,24,25,26,27]. E119G, R152K and R292K variants with reduced susceptibility to zanamivir could also be generated in the influenza B background in vitro [80].

#### 2.3.2. Oseltamivir

##### Resistance in Influenza A Viruses

H274Y is the most predominant mutation in oseltamivir-resistant clinical strains, such as seasonal A(H1N1), A(H1N1)pdm09 and highly pathogenic A(H5N1) strains, whilst R292K with E119V are the most common in N2 subtype viruses [32,41,75,76,81,82]. In oseltamivir-resistant A(H1N1)pdm09 isolates, I222R/K/V, S246N, and I117V, together with H274Y, were shown to have a synergistic effect on resistance [32,35,38,78,83,84]. I222V was capable of restoring NA affinity and activity reduced by H274Y. S246N and H274Y double mutations have been found in immunodeficient patients treated with oseltamivir. S246N reduced oseltamivir sensitivity six-fold and zanamivir sensitivity three-fold. These resistant strains have also been shown to retain their transmissibility [85]. R292K in the N2 subtype has been shown to have a greater effect on binding of oseltamivir and peramivir compared to other NA inhibitors [86].

##### Resistance in Influenza B Viruses

The majority of NAI-resistant influenza B viruses shows oseltamivir resistance associated with the following mutations in clinical specimens: K152N, D197N/E, A200T, I221T, and H273Y (B lineage specific numbering) [22,23,24,25,26,27]. Four influenza B viruses with I222T conferring reduced sensitivity to oseltamivir were recovered in Mainland China, during the 2010 and 2011 influenza seasons [51]. R292K has also been associated with reduced susceptibility to oseltamivir in vitro [80].

#### 2.3.3. Peramivir

##### Resistance in Influenza A Viruses

Similar to zanamivir, peramivir contains a guanidino group. It also has a hydrophobic group similar to that of oseltamivir. Therefore, mutations that affect the activity of oseltamivir and zanamivir can also affect peramivir activity. Peramivir resistance is mainly conferred by the H274Y substitution in seasonal A(H1N1) and A(H3N2) and A(H1N1)pdm09 viruses [38].

##### Resistance in Influenza B Viruses

Those influenza B viruses showing resistance to oseltamivir also demonstrate resistance to peramivir in both B/Yamagata and B/Victoria lineages with similar NA substitutions overall observed in these viruses [22,23,24,25,26,27]. An influenza B virus with the H274Y mutation was recovered from a patient with no known history of NAI treatment [87]. In vitro, E119D/A/V/G, R152K, and R292K mutations were shown to reduce susceptibility to peramivir [80].

#### 2.3.4. Laninamivir

##### Resistance in Influenza A Viruses

Laninamivir-resistant mutations have been reported in A(H1N1)pdm09 isolates, in the absence of laninamivir treatment. E119D/G in A(H1N1)pdm09 variants isolated from zanamivir-treated patients conferred highly reduced inhibition [30,31,32]. In vitro studies also demonstrated that mutations conferring resistance to other NAIs also conferred resistance to laninamivir. E119E/V/G/D conferred resistance in N3, N5, N6, N7, and N9 subtypes; R292K in N4, N6, N7, and N8 subtypes; I427L in N4; and Q136K in N8 and N9 subtypes [88]. However, H274Y, which can confer resistance to oseltamivir, did not confer resistance to laninamivir [88].

##### Resistance in Influenza B Viruses

Resistance to laninamivir in influenza B viruses is relatively uncommon, but resistance to B/Victoria- and B/Yamagata-lineage viruses was found in the 2013–2014, 2015–2016, and 2017–2018 seasons. Resistance was associated with R152K, G104E, H134N, I221T, and E117G (B lineage specific numbering) [22,23,24,25,26,27]. In vitro, E119G and Q140R have also been shown to confer resistance to laninamivir in influenza B viruses [46,48,52].

## 3. Hemagglutinin Inhibitors

HA is synthesized as a precursor protein, HA0, which is fusion incompetent, and assembles as a homotrimer on the viral membrane. Proteolytic cleavage by host trypsin-like proteases in the respiratory tract converts HA0 into the fusion-competent form containing the HA1 and HA2 subunits linked by a disulfide bond [89]. Each HA monomer consists of a globular head, stem, transmembrane, and cytoplasmic tail domains. Influenza viruses attach to sialic acids via the receptor binding domain on the globular head, located in HA1 after cleavage. The residues mediating this interaction are highly conserved and consist primarily of Y98, W153, H183, L194, and Y195 (H3 numbering, used throughout) [90,91]. After binding the virion is internalized into the cell in an endosome. The low pH of the endosome, which is acidified by the M2 ion channel, causes a conformational change in the HA2 subunit leading to exposure of the fusion peptide located at the N-terminus of HA2, mediating fusion of the endosomal and viral membranes. This leads to a fusion pore and the release of the viral gene segments into the cell. The critical role of HA in the viral lifecycle and its exposure on the virion make it an important antiviral target for vaccine-induced immune responses, monoclonal antibody therapies and structurally diverse small-molecule inhibitors. However, the selective pressure placed on HA means that it is the most variable protein on the virus. Further, unlike the NAIs, resistance to HA-targeted interventions often does not reduce viral fitness. Thus, HA-mediated resistance is a significant problem that complicates such interventions.

### 3.1. HA Inhibitors—Mechanisms of Action and Resistance

**Figure 3:**
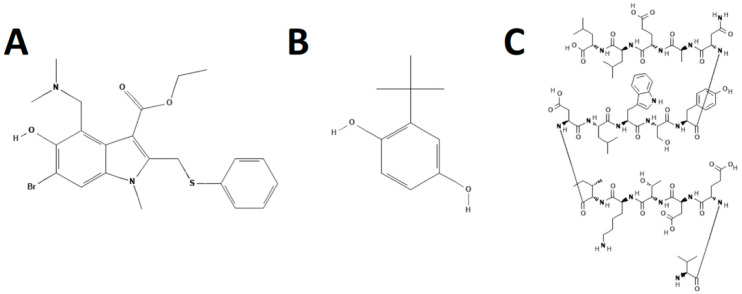
Structures of hemagglutinin fusion inhibitors. Umifenovir (Arbidol) (**A**), Tert-butyl hydroquinone (TBHQ) (**B**), and Flufirvitide-3 (**C**). Structures were adapted from PubChem.

Small molecule inhibitors targeting HA have not been developed or used clinically to the same degree as NAIs. The modes of action of HA inhibitors are to interfere with HA binding or fusion; therefore, these inhibitors target the globular head, containing the receptor binding domain, or the stalk, containing the fusion peptide, respectively (Figure 3).

#### 3.1.1. Umifenovir

The broad-spectrum Umifenovir (Arbidol) (DB13609) was the first drug used clinically to inhibit HA-induced viral-cell-membrane fusion [92]. It is currently available as an over-the-counter drug in Russia and China for prophylaxis and treatment of influenza. It is in Phase III and IV clinical trials in the USA. It is active against type A, B, and C influenza viruses, as well as other RNA and DNA viruses [92,93,94,95,96].

Arbidol is a fusion inhibitor, but it also exhibits a more general effect in perturbing membranes that likely contributes to its broad-spectrum antiviral activity. It binds to the hydrophobic pocket at the interface at the HA trimers in the stem domain. This increases the stability of the trimer, increasing the pH of fusion and preventing the low pH-induced HA from converting to its fusogenic state. As a result, viral fusion is inhibited, preventing genome release and ultimately blocking infection [92,97]. In vitro, Arbidol has a high barrier to resistance, with resistance mutations appearing after 15 passages. This is compared to the low barrier of resistance of the M2 inhibitors, where resistance mutations occur with as little as two to three passages in the presence of M2 inhibitors [98]. K51N, K117R, Q27N, and Q42H, in the HA2 subunit, have been identified to confer resistance to Arbidol in vitro [98]. However, no incidences of resistance have been documented in clinical trials or during clinical use thus far.

#### 3.1.2. Newer HA Fusion Inhibitors

Recently, several compounds have shown promising in vitro activity as HA fusion inhibitors. Tert-butyl hydroquinone (TBHQ) is a small-molecule compound that, like Arbidol, binds to the HA stalk at a binding site that partially overlaps that of Arbidol and interferes with viral fusion. The result is a cross-linking of the three HA monomers in the glycoprotein spike, thus inhibiting conformational changes necessary for membrane fusion. Since the mechanism of action of TBHQ is similar to that of Arbidol, it is likely that a high barrier to resistance also exists, although this has yet to be determined.

Indole-substituted spirothiazolidinones are another type of small molecule compound with overlapping binding pockets with Arbidol and TBHQ but improved potency against A(H3N2) viruses [99]. Several other fusion inhibitors have also demonstrated activity in vitro, including RO5464466, BMY-27,709, Stachyflin, and MBX2329. However, subtype dependency of their action has limited further development [100,101,102,103,104].

The HA stalk, being more conserved than the head domain, has been studied as a target for broadly neutralizing antibodies. Based on the molecular interactions of these antibodies with stalk epitopes, a small molecule, JNJ4796, was identified that mimics the interactions of broadly neutralizing stalk antibodies with HA and inhibits the pH dependent conformational change of the HA2 of Group 1 HAs but is orally active, unlike antibodies [105]. Experiments in vitro and in vivo yielded promising results, including protecting mice from lethal infections with a A(H1N1)pdm09 influenza virus.

#### 3.1.3. Peptide Fusion Inhibitors

Flufirvitide-3 is a 16 amino acid peptide derived from the fusion initiation region of HA2 that is inhibitory to subtype H1, H3, H5, and influenza B viruses in vitro and in ferrets [106,107]. It is administered as a nasal inhalant and is now being assessed in a Phase II clinical trial in the USA. P155-185-chol is another peptide-based fusion inhibitor based on the C-terminus of the HA protein of a H7N9 influenza virus [108]. This peptide inhibited fusion of H7N9 influenza virus but not a subtype H5 virus, indicating possible subtype dependency.

#### 3.1.4. Receptor Binding Site Inhibitors

HA receptor binding site inhibitors aim to block the interaction between HA and sialic acids, thus preventing infection. Multivalent sialic acid mimics, designed to out-compete the binding of HA with sialic acids, include sialylglycopolymers, dendritic sialosides, and sialic acid–containing liposomes [109,110]. Icosahedral bacteriophages have also been used as multivalent binders that present sialic acid ligands in such a configuration to “coat” influenza viruses, preventing their attachment to cells [111]. Peptides that bind to the HA head domain have also been developed as sialic acid–mimics, to block the HA–sialic acid interaction and have in vitro activity against A(H1N1) and A(H3N2) influenza viruses [112]. Nucleic acids designed to bind to the HA globular head are also capable of blocking the interaction with sialic acid, both in vitro and in vivo [113,114].

## 4. Therapeutic Strategies to Combat Drug Resistance

Combinations of NAIs or NAIs and other antiviral agents have been assessed as part of possible therapeutic strategies for patients infected with NAI-resistant influenza viruses or as strategies to mitigate or slow the emergence of resistant viruses. Whilst zanamivir combined with oseltamivir did not yield any clear benefits in terms of clinical outcome or in counteracting the development of resistance [115,116,117], oseltamivir combined with convalescent plasma or hyperimmune globulin has been associated with reduced mortality compared to oseltamivir alone. Oseltamivir, in combination with sirolimus and corticosteroids as anti-inflammatories to prevent tissue damage, was also associated with reduced mortality in critically ill patients [118,119]. A triple combination of amantadine, oseltamivir, and ribavirin, a synthetic guanosine analogue, was tested in a Phase I study in immunocompromised influenza patients. This treatment regimen showed some effect in reducing viral loads and was well tolerated, but there were no significant improvements in clinical outcomes, as compared to oseltamivir treatment alone [120,121]. Overall, whilst combination therapies show some indications of efficacy, more studies are needed to ascertain potential benefits for reducing the likelihood of resistance and improving clinical outcomes.

## 5. Discussion

Influenza remains a significant threat to global public health. One of the important factors behind this threat is the unpredictability of influenza viruses. Even with global networks monitoring the circulation of viruses in humans and animals, it is not possible to predict with accuracy the emergence of new viruses that could have the potential to cause outbreaks or even pandemics. It is in a rapidly evolving situation such as an epidemic or pandemic where antivirals play a particularly important role. However, the anti-influenza repertoire has for decades been largely restricted to one or two drugs against one target, NA. Therefore, drug-resistant strains pose a serious threat to public health. Fortunately, the incidence of NAI resistance remains low for the time being. Further, more antivirals are gaining approval for widespread use and are proving to be effective, including those against other viral targets such as HA. Whilst the globular head of HA, particularly proximal to the active site, is one of the most variable on the virus, the HA stalk domain is much more conserved, although it is not as exposed or accessible as the globular head. However, its critical role in membrane fusion makes it an important target. Fusion inhibitors such as Arbidol are small-molecule compounds and can more readily access the stalk domain, meaning they can interfere with this important step. These fusion inhibitors show significant promise as broad-spectrum antivirals and are likely to become more widely used. Adding to the diversity of the antiviral repertoire means that the emergence of resistance against any one antiviral becomes less concerning and provides for a more robust response to emerging viruses, thus slowing the spread of outbreaks and reducing their public health impact. In that regard, antivirals remain a critical component to the public health interventions currently available against influenza viruses.

## Figures and Tables

**Table 1:** Recommended therapeutic regimens for neuraminidase inhibitors.

Neuraminidase Inhibitor	Route	Therapeutic Regimen			Peak Time (Hours)	Half-Life (Hours)	Clearance
		Frequency	Age/ Weight	Dose			
Zanamivir	Inhalation	twice daily for 5 days	≥7 years old	10 mg	1–2	2	Urine within 24 h or feces (unabsorbed)
Oseltamivir	Oral	twice daily for 5 days	≤15 kg	30 mg	3–4	6–10	Renal excretion
			16–23 kg	45 mg			
			24–40 kg	60 mg			
			>40 kg	75 mg			
			≥13 years	75 mg			
Peramivir	Intravenous	Once daily for 5 to 10 days	Adult	600 mg	2–4	7.7–20.8	Renal excretion (90%)
Laninamivir	Inhalation	Once	≤10 years	20 mg	4	67	Renal excretion (34%)
			≥10 years	40 mg			

**Table 2:** Summary of common neuraminidase mutations conferring neuraminidase inhibitor resistance identified in clinical isolates.

Influenza Type/Subtype	Neuraminidase Inhibitor	Common Substitution in Neuraminidase (N2 Numbering)	References
A(H1N1)pdm09	Zanamivir	E119D/G and S246R	[23,25,26,27,29,30,31,32,33,34,35,36]
	Oseltamivir	E119D, I222R, S246G/R and H274Y	
	Peramivir	E119D/G, S246R and H274Y	
	Laninamivir	E119D/G and S246R	
A(H5N1)	Zanamivir	None	[23,37,38]
	Oseltamivir	H274Y and N294S	
	Peramivir	H274Y	
	Laninamivir	None	
A(H3N2)	Zanamivir	E119I	[12,39,40,41,42,43,44,45]
	Oseltamivir	E119I/V, R292K and N294S	
	Peramivir	R292K	
	Laninamivir	None	
B	Zanamivir	R152K, I222L and G404S	[26,36,41,46,47,48,49,50,51,52]
	Oseltamivir	R152K, D198E, I222L and N294S	
	Peramivir	E107K, R152K, D198E, I222L/T and N294S	
	Laninamivir	R152K	

## Data Availability

Not applicable.
